# Proteomic Profiling Reveals Intracellular Regulatory Network Disturbance Induced by QseB Deletion in *Glaesserella parasuis*

**DOI:** 10.3390/microorganisms14092073

**Published:** 2026-09-16

**Authors:** Xuefeng Yan, Congwei Gu, Mingde Zhao, Lvqin He

**Affiliations:** 1 School of Physical Education, Southwest Medical University, Luzhou 646000, China; 2 Experimental Animal Center, Technology Department, Southwest Medical University, Luzhou 646000, China; 3 Luzhou Key Laboratory of Model Animal and Human Disease Research, Southwest Medical University, Luzhou 646000, China

**Keywords:** *G. parasuis*, QseB, scanning electron microscopy, transmission electron microscopy, DIA quantitative proteomics

## Abstract

The highly conserved QseB/QseC two-component system (TCS) in *Glaesserella parasuis* (*G. parasuis*) regulates gene expression, with QseC as a histidine kinase and QseB as a response regulator. This study employed data-independent acquisition (DIA) quantitative proteomics to investigate the effects of *qseB* deletion on bacterial morphology, proteome, and molecular regulation. Electron microscopy revealed that the Δ*qseB* mutant exhibited ultrastructural damage, including cell shrinkage, membrane impairment, cell wall separation, and protoplast dissolution, indicating that the loss of *qseB* resulted in severe compromise of cellular structural integrity. A total of 1493 proteins were identified by DIA quantitative proteomics. Fifty-five proteins were differentially expressed between the Δ*qseB* mutant and the wild-type strain SC1401. Among them, 24 were upregulated and 31 were downregulated. Upregulated proteins were mainly heat shock proteins (DnaK, HslV/U, ClpB), molecular chaperones (GroL, HtpG, HslO), and co-chaperone GroES, along with translation inhibitors RaiA and RsfS. These proteins participate in stress response, protein folding, and proteostasis maintenance. The downregulated proteins included QseC, LolB, CarB, ThiL, and Tgt, which are linked to TCSs, quorum sensing, and transport functions. KEGG analysis indicated that the differentially expressed proteins are involved in RNA degradation, amino acid metabolism, and ABC transporters. Protein–protein interaction (PPI) network analysis identified 10 core hub proteins, including GroEL, HslU, and QseC. These proteins may play key roles in maintaining cellular homeostasis. Integrated multi-omics analysis identified three genes (*dnaK*, *hslU*, *hslO*) that were differentially expressed in both transcriptomic and proteomic data. qRT-PCR results confirmed these findings. In summary, loss of *qseB* disrupts the QseB/QseC system. This affects stress response, metabolism, and cell structure. These changes may affect bacterial fitness and host adaptation, providing clues for understanding the pathogenic mechanisms of *G. parasuis*.

## 1. Background

Glässer’s disease is an infectious condition affecting weaned and growing pigs caused by *G. parasuis* [1,2]. The disease primarily manifests as sepsis in weaned pigs and can occur as early as three weeks of age. Key stressors such as dietary changes, weaning, transportation, and sudden climate variations are major contributing factors [3]. Clinical studies indicate that bacterial infections, including *G. parasuis*, *Streptococcus suis*, and *Actinobacillus pleuropneumoniae,* are often exacerbated by co-infection with porcine reproductive and respiratory syndrome (PRRS) virus. For specific pathogen-free (SPF) pigs or high-health herds, vaccination is recommended to prevent disease outbreaks. However, because *G. parasuis* comprises as many as 15 serotypes, vaccines can induce serotype-specific protection and partial cross-protection; they cannot cover all heterologous serotypes, and the protective efficacy against different strains within the same serotype remains inconsistent [4]. Research on *G.parasuis* infection has continued to advance. Although key aspects such as its virulence factors, immune protection mechanisms, pathogenic processes, host interactions, and immune evasion strategies have not yet been fully elucidated, the growing understanding of common bacterial pathogenic mechanisms has laid a foundation for developing efficient and low-cost attenuated vaccines and antibacterial agents. This progress also offers new insights for the clinical management of upper respiratory tract infections caused by *G. parasuis* in pigs.

Bacteria sense external stimuli through two-component systems (TCSs) and mount adaptive responses to adjust to new environments, thereby promoting survival and reproduction. Multiple TCSs are typically present in bacteria, which may occur as paired histidine kinase/response regulator (HK/RR) complexes, or as orphan HKs or orphan RRs. The QseB/QseC two-component system is a well-known virulence regulator in Enterobacteriaceae and Pasteurellaceae families [5]. In *Actinobacillus pleuropneumoniae* (APP), QseB/QseC is linked to bacterial cell division, while in *Haemophilus influenzae*, it participates in biofilm formation [6]. In *Escherichia coli*, QseBC regulates bacterial flagella and motility [7]. Additionally, QseB/QseC contributes to host cell invasion and virulence in *Salmonella* [8]. Our previous QseC multi-omics studies [9,10] revealed that QseC deletion affects membrane integrity, lipopolysaccharide (LPS) biosynthesis, and glycerolipid metabolism. However, the proteomic landscape regulated by QseB, the cognate response regulator, has not been systematically analyzed. Therefore, this study was designed to address this gap by comparing the proteome of the Δ*qseB* mutant with that of the wild-type strain.

Phosphorylation of the response regulator protein QseB can occur either in a QseC-dependent manner or independently [11]. QseC perceives external signals such as autoinducer-3 (AI-3), adrenaline, or noradrenaline, undergoes autophosphorylation, and subsequently transfers the phosphate group to its regulatory protein QseB. While QseC is a membrane-bound histidine kinase that senses external signals, QseB is a cytoplasmic response regulator that directly binds DNA to modulate gene expression [5]. Thus, the two components of this system are expected to control partially overlapping but distinct regulatory networks. Activated QseB plays an important role in bacterial virulence, flagellar assembly, and biofilm formation by regulating the expression of genes related to bacterial motility and pathogenicity [12,13]. In our preliminary study, we identified a wild-type strain of *G. parasuis*, SC1401, exhibiting high natural transformation efficiency, and constructed Δ*qseB* and Δ*qseBC* mutant strains using natural transformation. We further observed that the QseBC TCS significantly modulates the virulence of *G. parasuis* in a mouse infection model [14].

In contrast to QseC, which acts as a membrane-bound sensor kinase, QseB is a cytoplasmic response regulator that directly controls downstream gene transcription. Although their functions may overlap in certain pathways, QseB likely possesses a unique regulatory network that has not yet been systematically investigated at the proteomic level. To further investigate the functional mechanism of QseB in *G. parasuis*, we conducted a comparative proteomic analysis of the parental strain SC1401 and the Δ*qseB* mutant under standard growth conditions. These proteomic data will provide valuable insights for system-wide modeling of signal transduction and transcriptional regulation, and establish an important foundation for further research into the role of QseB in the infection process, pathogenic mechanisms, and control strategies related to *G. parasuis*.

## 2. Materials and Methods

### 2.1. Cultivation Conditions for Bacterial Strains

Bacterial culture conditions were as described previously [15]. The *G. parasuis* parent strain SC1401 was provided by the Pig Disease Research Center of Sichuan Agricultural University (Ya’an, China), and the Δ*qseB* mutant strain was constructed and maintained in our laboratory. All strains, including both wild-type and mutant variants, were cultured on Tryptic Soy Agar (TSA, Cat. No. 236950, BD Difco, Sparks, MD, USA) or in Tryptic Soy Broth (TSB, Cat. No. 211825, BD Bacto, Sparks, MD, USA) medium, supplemented with 0.1% (*w*/*v*) nicotinamide adenine dinucleotide (NAD, Sigma-Aldrich, St. Louis, MO, USA) and 5% heat-inactivated newborn calf serum (Solarbio, Beijing, China). For simplicity, TSA and TSB containing these supplements are referred to as TSA^++^ and TSB^++^, respectively.

### 2.2. Morphological Observation of ΔqseB Mutant Strains

Scanning electron microscopy (SEM) and transmission electron microscopy (TEM) were performed as previously described [10]. The effect of *qseB* deletion on the morphology of *G. parasuis* was examined using SEM and TEM. After overnight culture, both the parental strain and the Δ*qseB* mutant were inoculated into fresh TSB^++^ liquid medium at a 1:100 ratio and cultured at 80 r/min for 24 h. Bacterial cells were collected by centrifugation at 4000× *g* for 10 min, washed with PBS, and recentrifuged. The pellet was then fixed with 2.5% glutaraldehyde at room temperature in the dark for 2 h. Subsequently, the fixed samples were processed following standard protocols. For SEM, samples were dehydrated through a graded ethanol series, critical-point dried, and sputter-coated with gold. For TEM, samples were post-fixed with 1% osmium tetroxide, dehydrated through a graded ethanol series, embedded in resin, and ultrathin-sectioned. Images were then acquired and analyzed by SEM and TEM.

### 2.3. Extraction of Total Bacterial Protein

Total protein extraction was performed following the protocol described in our recent QseC proteomic study. Briefly, the parental strain SC1401 and the Δ*qseB* mutant strain of *G. parasuis* were inoculated into 3 mL of TSB^++^ medium and cultured with shaking at 37 °C and 220 rpm. Each strain was also streaked onto TSA^++^ plates and incubated overnight at 37 °C. The next day, a single colony was selected and inoculated into TSB^++^ medium, followed by incubation at 37 °C with shaking at 220 rpm until the OD_600_ reached 1.6. Bacterial cells were harvested by centrifugation, and the pellet was stored at −80 °C for further use. The frozen bacterial cells were ground into a fine powder under low-temperature conditions and quickly transferred to a centrifuge tube pre-cooled in liquid nitrogen. An appropriate amount of SDT lysis buffer containing 100 mM NaCl and dithiothreitol (DTT) at a 1:100 dilution was added, and the mixture was vortexed thoroughly. The samples were sonicated in an ice-water bath for 5 min to ensure complete cell lysis. They were then heated at 95 °C for 8–15 min, followed by incubation in an ice bath for 2 min. After centrifugation at 4 °C and 12,000× *g* for 15 min, the supernatant was collected. A sufficient volume of iodoacetamide (IAM) solution was added, and the reaction was carried out in the dark for 1 h. Four volumes of acetone pre-cooled to −20 °C were added, and the mixture was precipitated at −20 °C for at least 2 h. The sample was then centrifuged at 4 °C and 12,000× *g* for 15 min to collect the protein precipitate. The precipitate was resuspended in 1 mL of pre-cooled acetone, washed, centrifuged again, and air-dried to obtain the total protein sample. Finally, an appropriate amount of dissolution buffer (DB buffer) was added to completely dissolve the protein precipitate. Three independent biological replicates were prepared for each strain (SC1401 and the Δ*qseB* mutant).

### 2.4. Vanquish™ Neo UHPLC-Astral Liquid DIA Method

Mobile phase A was water containing 0.1% formic acid, and mobile phase B was 80% acetonitrile containing 0.1% formic acid. The lyophilized powder was dissolved in 10 µL of mobile phase A and centrifuged at 14,000× *g* for 20 min at 4 °C. An aliquot of the supernatant containing 200 ng of protein was injected for analysis. The analysis was performed using a Vanquish Neo ultra-high-performance liquid chromatography (UHPLC) system coupled to a Thermo Orbitrap Astral mass spectrometer (Thermo Fisher Scientific, Bremen, Germany) equipped with an Easy-Spray (ESI) ion source. Data were acquired in data-independent acquisition (DIA) mode, with an MS1 full scan range of *m*/*z* 380–980 at a resolution of 240,000 (at *m*/*z* 200), and an MS2 scan range of *m*/*z* 150–2000 at an Astral resolution of 80,000 and a maximum injection time of 3 ms. Raw mass spectrometry data were collected in .raw format.

### 2.5. Protein Identification and Functional Analysis of Differentially Expressed Proteins (DEPs)

The raw mass spectrometry data (.raw files) were searched against the UniProt protein database using DIA-NN 1.8.1 software. To enhance identification reliability, the results were filtered to retain only peptides and proteins with a peptide-spectrum match confidence > 99%, followed by false discovery rate (FDR) filtering to remove those with an FDR > 1%. All proteomic experiments were performed (*n* = 3). Protein abundance values from biological replicates were used for group comparison, and statistical significance was assessed using Student’s *t*-test. The *p*-values were adjusted for multiple testing using the Benjamini–Hochberg method.

To determine the appropriate cutoff for differentially expressed protein screening, we compared the number of DEPs identified at different fold-change (FC) thresholds. A stricter threshold of FC > 1.5 yielded only 7 upregulated and 10 downregulated proteins, while FC > 1.2 combined with *p* < 0.05 identified 24 upregulated and 31 downregulated proteins, providing broader coverage of biologically relevant changes. Therefore, proteins with FC > 1.2 (upregulated) or <0.83 (downregulated) and *p* < 0.05 were considered significantly differentially expressed.

Functional annotation of identified proteins was performed using InterProScan v5.22-61.0 to obtain Gene Ontology (GO) terms and InterPro (IPR) domain assignments, while functional classification and pathway annotation were conducted using the Clusters of Orthologous Groups (COG) and Kyoto Encyclopedia of Genes and Genomes (KEGG) databases. Differentially expressed proteins (DEPs) were visualized through volcano plot and cluster heatmap analyses, and further subjected to enrichment analyses based on GO, IPR, and KEGG pathways. Potential protein–protein interactions among DEPs were predicted using the STRING database.

### 2.6. Quantitative Real-Time Reverse Transcription-PCR (qRT-PCR) Analysis

The wild-type strain SC1401 and the Δ*qseB* mutant strains were cultured under the same conditions to an OD_600_ of approximately 0.8. Total bacterial RNA was extracted and reverse-transcribed to cDNA. With 16S rRNA as an internal reference gene, qRT-PCR was performed to quantify the expression levels of four randomly selected differentially expressed genes to validate the DIA proteomics data. To ensure representative validation, these four genes (*qseC*, *malG*, *YdeI*, and *thiM*) were selected to cover both upregulated and downregulated expression trends and diverse functional categories (signal transduction, transport, and metabolism). Primer sequences are listed in Table 1.

### 2.7. Correlation Analysis of the Transcriptome and Total Proteome

Integrative analysis of transcriptomic and proteomic data can help uncover post-transcriptional regulatory mechanisms of gene expression. In this study, proteomic data [16]. This multi-omics analysis was designed to systematically examine relevant biological processes and to explore potential regulatory relationships between the transcriptomic and proteomic levels. This transcriptomic dataset [17] was generated using the same bacterial strains (*G. parasuis* SC1401 and the Δ*qseB* mutant) and growth conditions (TSB^++^ medium at 37 °C) at the same growth phase (OD_600_ ≈ 0.8), ensuring comparability between the two omics datasets.

### 2.8. Data and Code Availability

The mass spectrometry data have been deposited in the ProteomeXchange Consortium with the dataset identifier PXD065386.

## 3. Results

### 3.1. Morphological and Ultrastructural Observation of ΔqseB Mutant

**Figure 1:**
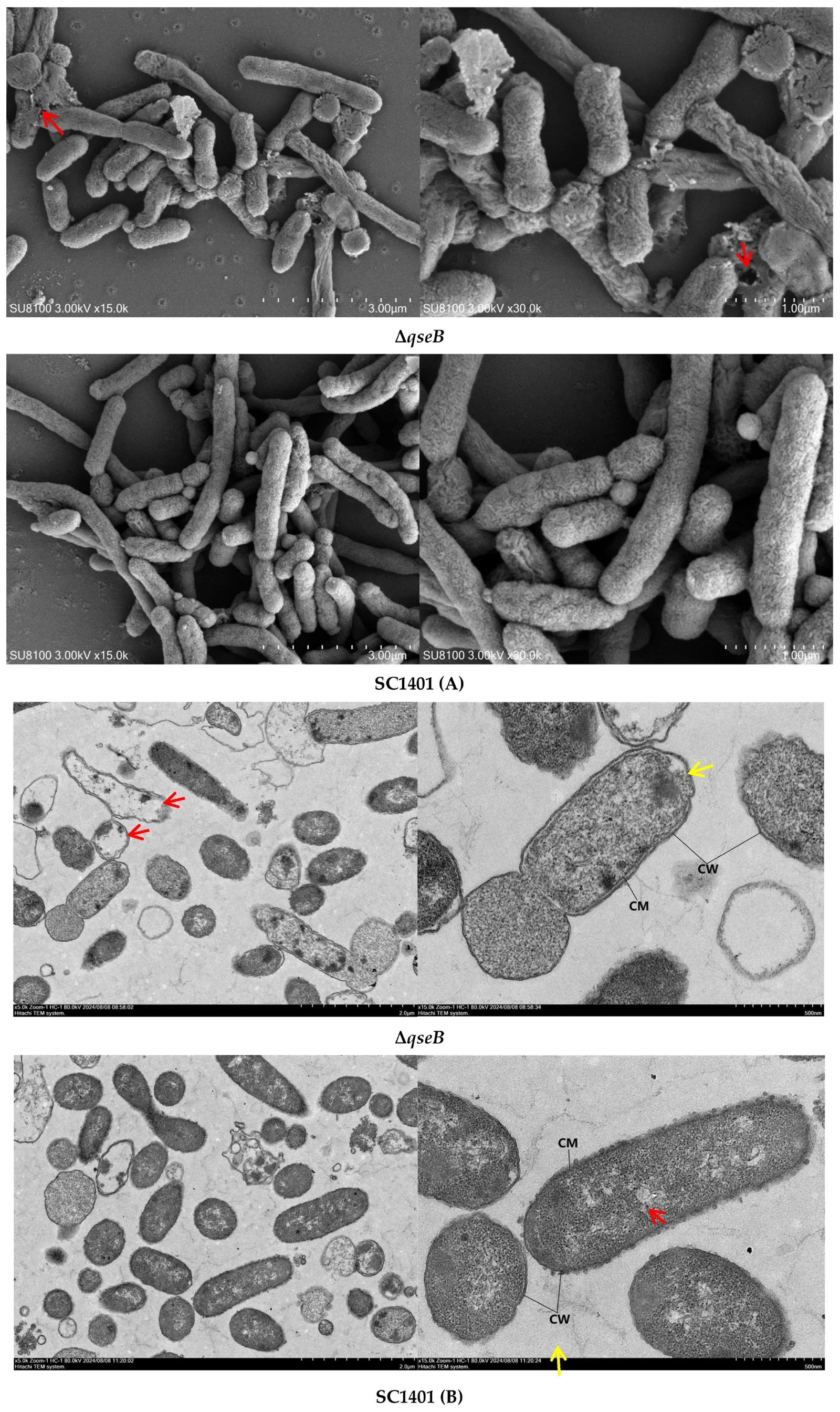
Observation of bacterial morphology and internal structure by electron microscopy. (**A**) SEM images reveal morphological differences between the wild-type and Δ*qseB* mutant strains. The Δ*qseB* mutant exhibits localized cell wall damage (red arrow). In contrast, SC1401 cells display intact cell walls and smooth surfaces. (**B**) TEM shows internal structures. Most Δ*qseB* cells exhibit significant damage and partial disintegration (red arrows). Small regions of dissolved or broken cell wall and cell membrane (yellow arrows) are also observed, with separation between the cytoplasm and cell wall. The cytoplasm appears sparse and electron-lucent due to dissolution. SC1401 cells retain intact cell walls and membranes, with no observed separation between the cytoplasm and cell wall. However, localized protoplast dissolution and cytoplasmic inhomogeneity are noted (red arrow).

The morphology and ultrastructure of the wild-type strain SC1401 and the Δ*qseB* mutant were examined by scanning electron microscopy (SEM; Figure 1A) and transmission electron microscopy (TEM; Figure 1B). SEM revealed marked morphological differences between the two strains. SC1401 cells were intact with smooth surfaces, whereas the Δ*qseB* mutant showed widespread wrinkling, partial rupture, and visible adherence of surface secretions or cellular debris. TEM further revealed severe ultrastructural damage in the Δ*qseB* mutant compared to the wild-type strain. Specifically, many Δ*qseB* cells exhibited disintegration, substantial protoplast dissolution, disruption of cell wall and membrane integrity, and apparent separation between the cytoplasm and cell wall. In contrast, SC1401 retained largely intact cellular envelopes with only minor, localized protoplast dissolution.

### 3.2. Protein Quantification and Differential Expression Analysis

**Figure 2:**
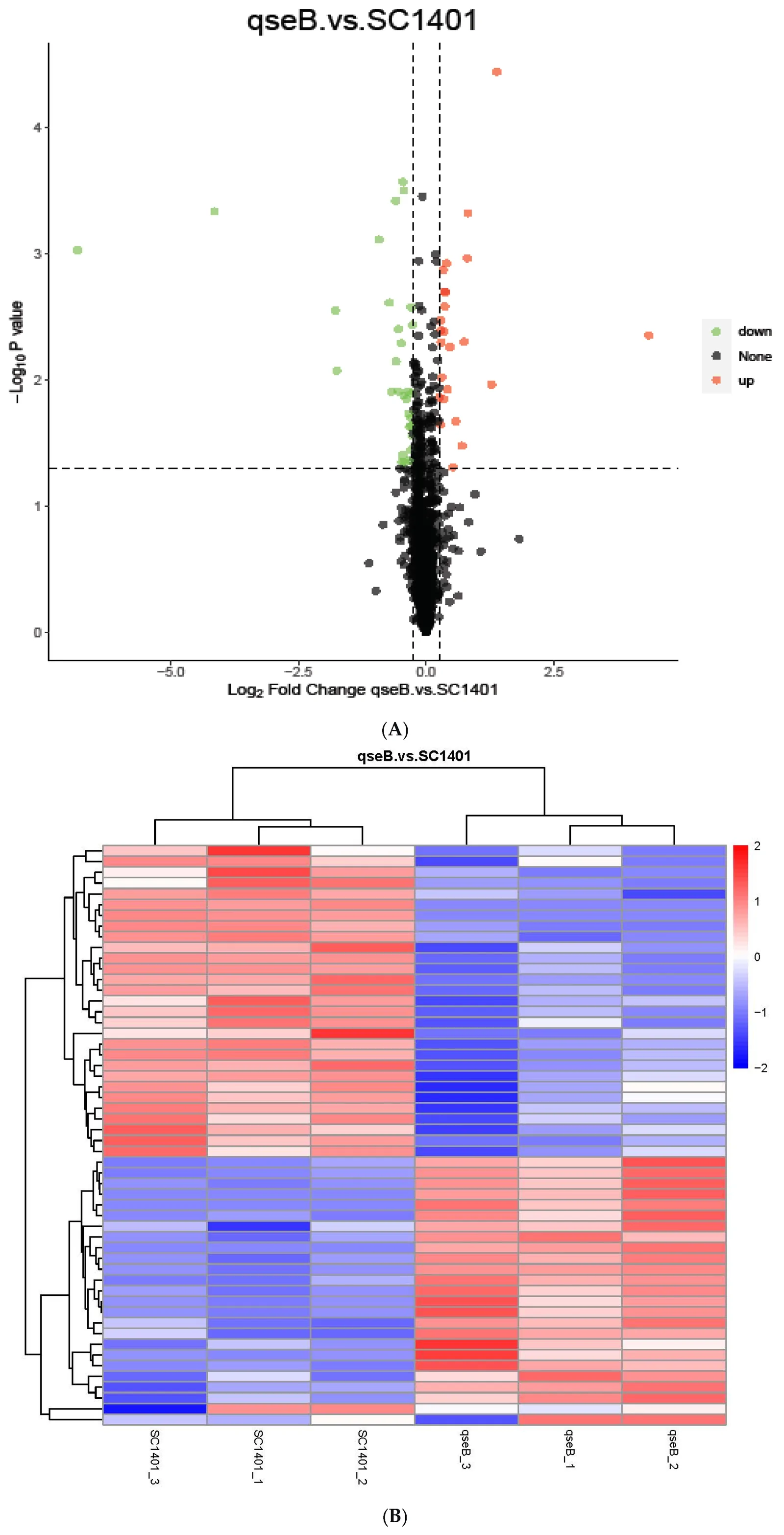
Differential protein analysis. (**A**) Volcano plot of differentially expressed proteins. The fold change in each protein was log_2_-transformed, and the corresponding *p*-value was plotted as −log_10_. The horizontal axis represents the log_2_ fold-change, and the vertical axis corresponds to the −log_10_ (*p*-value). Black dots indicate proteins with no significant differential expression, red dots indicate upregulated proteins, and green dots indicate downregulated proteins. (**B**) Clustered heatmap of differentially expressed proteins. Hierarchical analysis was performed based on the relative abundance of differentially expressed proteins across samples. Each row represents a protein normalized by Z-score transformation, calculated as (observed value–row mean)/row standard deviation. Vertical clustering denotes sample groupings, and horizontal clustering represents protein groupings. Shorter branches indicate higher similarity. The vertical clustering reveals distinct patterns of protein expression among sample groups.

A total of 20,122 peptides and 1493 proteins were identified (Table S1). Principal component analysis (PCA) was performed to assess overall differences in protein expression among samples and variability within groups, and the coefficient of variation (CV) was used to evaluate data dispersion and repeatability (Figure S1A,B). Using a fold change (FC) > 1.2 and *p* < 0.05, we identified 24 upregulated and 31 downregulated proteins among the 1489 proteins detected in both the SC1401 and Δ*qseB* groups (Figure 2A, Table S2). The upregulated proteins included RaiA, DnaK, LeuA, HslV, HtpG, HslO, HslU, RsfS, RluA, GroEL, RlmE, and ClpB. Downregulated proteins included LolB, YkgO, Tgt, ThiL, QseC, and CarB. Detailed information on all differentially expressed proteins is provided in Table S3. Hierarchical clustering was then applied to identify protein clusters with similar expression patterns. The resulting clusters are shown in Figure 2B, and the corresponding data are detailed in Table S4.

### 3.3. Functional Characterization and KEGG Pathway Enrichment Analysis of Differentially Expressed Proteins

**Figure 3:**
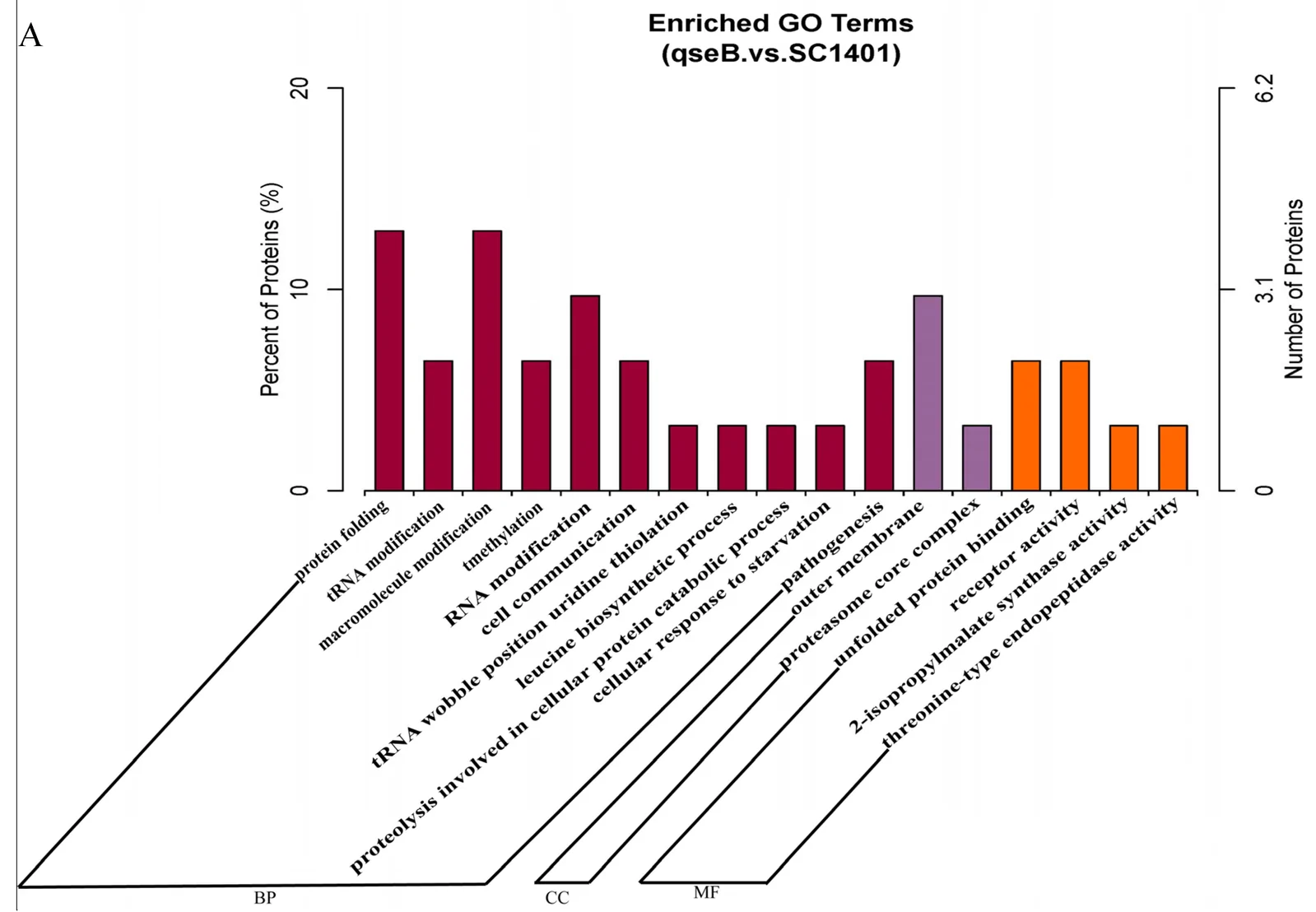

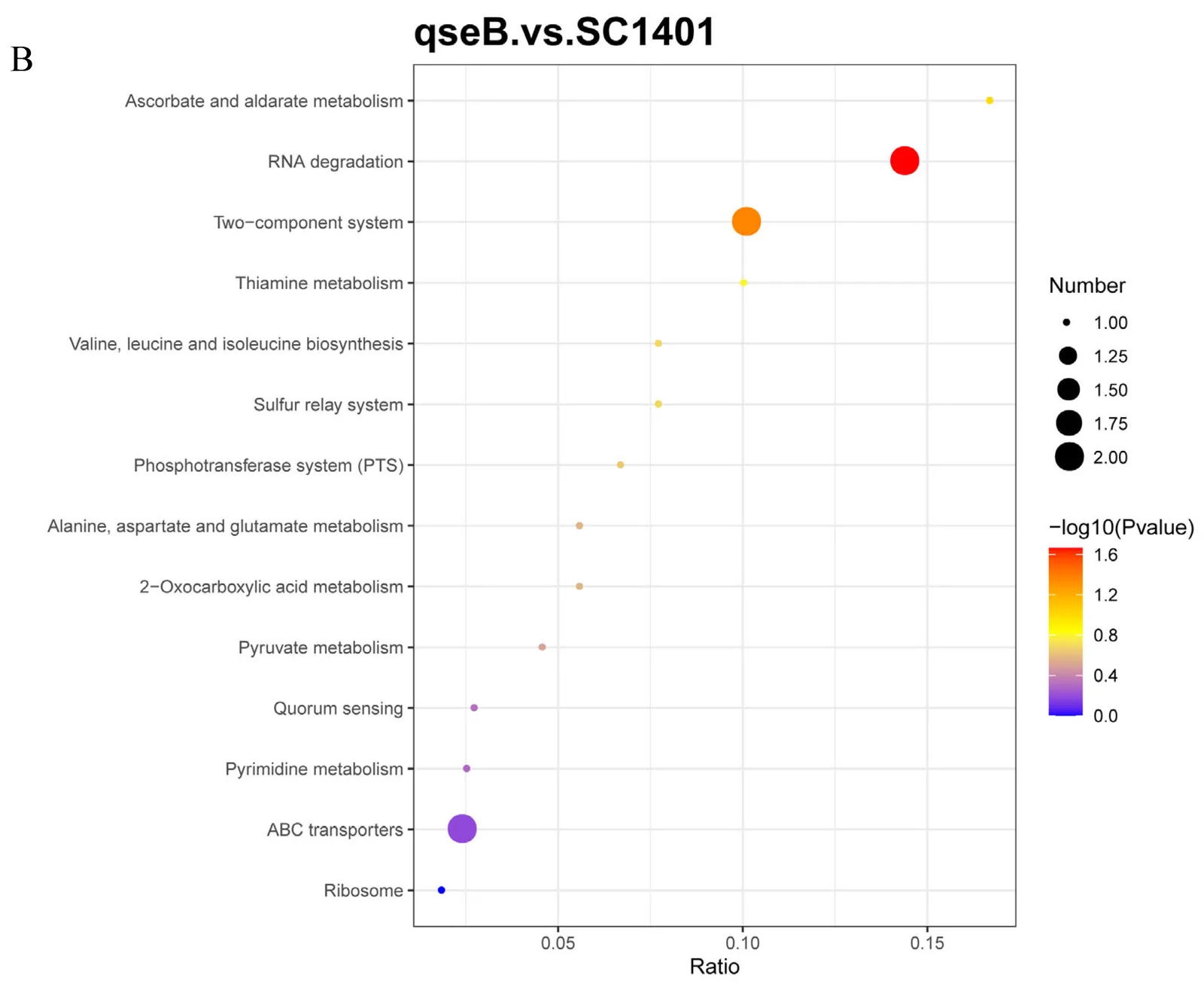
Differential protein enrichment analysis. (**A**) GO enrichment bar chart. The figure shows enrichment results across three major categories, with a maximum of 20 terms per category (*p* < 0.05). The y-axis percentage represents the ratio x/n, as indicated in the accompanying table. (**B**) KEGG enrichment scatter plot. The horizontal axis represents the ratio of differentially expressed proteins to the total number of identified proteins in a given pathway. A larger value indicates a higher enrichment of differentially expressed proteins in that pathway. Dot color corresponds to the *p*-value from the hypergeometric test, with a gradient from blue (higher *p*-value) to red (lower *p*-value). More intense red indicates greater statistical significance. Dot size reflects the number of differentially expressed proteins in the corresponding pathway, with larger dots representing more proteins.

Gene Ontology (GO) annotation revealed that the upregulated proteins in the Δ*qseB* mutant were primarily associated with protein folding, methylation, the phosphoenolpyruvate-dependent sugar phosphotransferase system, stress response, binding, and integral membrane components (Table S5). The downregulated proteins in the Δ*qseB* were mainly involved in receptor activity, cell communication, cellular response to starvation, sequence-specific DNA binding, membrane association, phosphorelay sensor kinase activity, cellular response to stimulus, signal transduction, pathogenesis, gene expression, transferase activity, transport, and various cellular processes (Figure 3A; Table S6). KEGG pathway enrichment analysis (Figure 3B) indicated that the upregulated proteins were significantly enriched in RNA degradation, ascorbate and aldarate metabolism, valine, leucine and isoleucine biosynthesis, the phosphotransferase system (PTS), 2-oxocarboxylic acid metabolism, pyruvate metabolism, amino acid biosynthesis, and microbial metabolism in diverse environments (Table S7). Conversely, the downregulated proteins were predominantly enriched in the two-component system, thiamine metabolism, sulfur relay system, alanine, aspartate and glutamate metabolism, ABC transporters, quorum sensing, pyrimidine metabolism, and ribosome-related pathways.

### 3.4. Construction and Module Analysis of the Protein–Protein Interaction Network of Differentially Expressed Proteins

**Figure 4:**
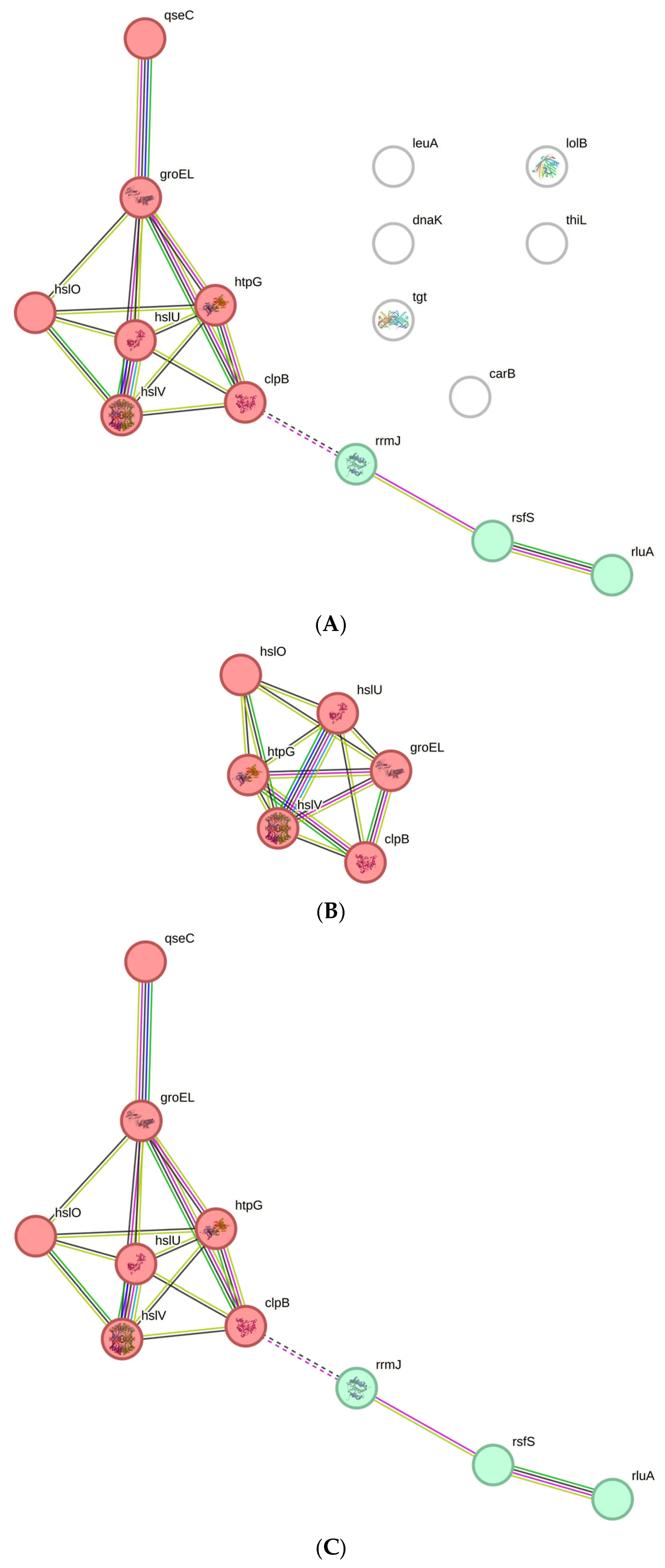
Analysis of significant protein modules and protein–protein interaction networks of differentially expressed proteins. (**A**) Protein–protein interaction (PPI) network of differentially expressed proteins. (**B**) Significant functional modules of differentially expressed proteins identified by MCODE. (**C**) Core protein interaction network extracted using cytoHubba. In each network, nodes represent individual proteins, and node size corresponds to the number of interacting partners. Larger nodes indicate a higher degree of connectivity.

A protein–protein interaction (PPI) network of differentially expressed proteins was constructed using Cytoscape 3.10.3, comprising 16 nodes and 18 edges (Figure 4A). A highly interconnected protein module was identified using the MCODE plugin in Cytoscape (Figure 4B). Proteins with high node connectivity were identified as hub proteins and prioritized for further analysis. The top 10 core proteins were identified using the cytoHubba plugin via the MCC algorithm, including GroEL, HslU, HtpG, HslV, ClpB, HslO, RsfS, RrmJ, QseC, and RluA (Table S8). These 10 hub proteins were subsequently reimported into the STRING database to construct a focused PPI network (Figure 4C), which revealed statistically significant interactions among the proteins (*p* = 1.14 × 10^−14^).

### 3.5. Validation of Proteomic Results

**Figure 5:**
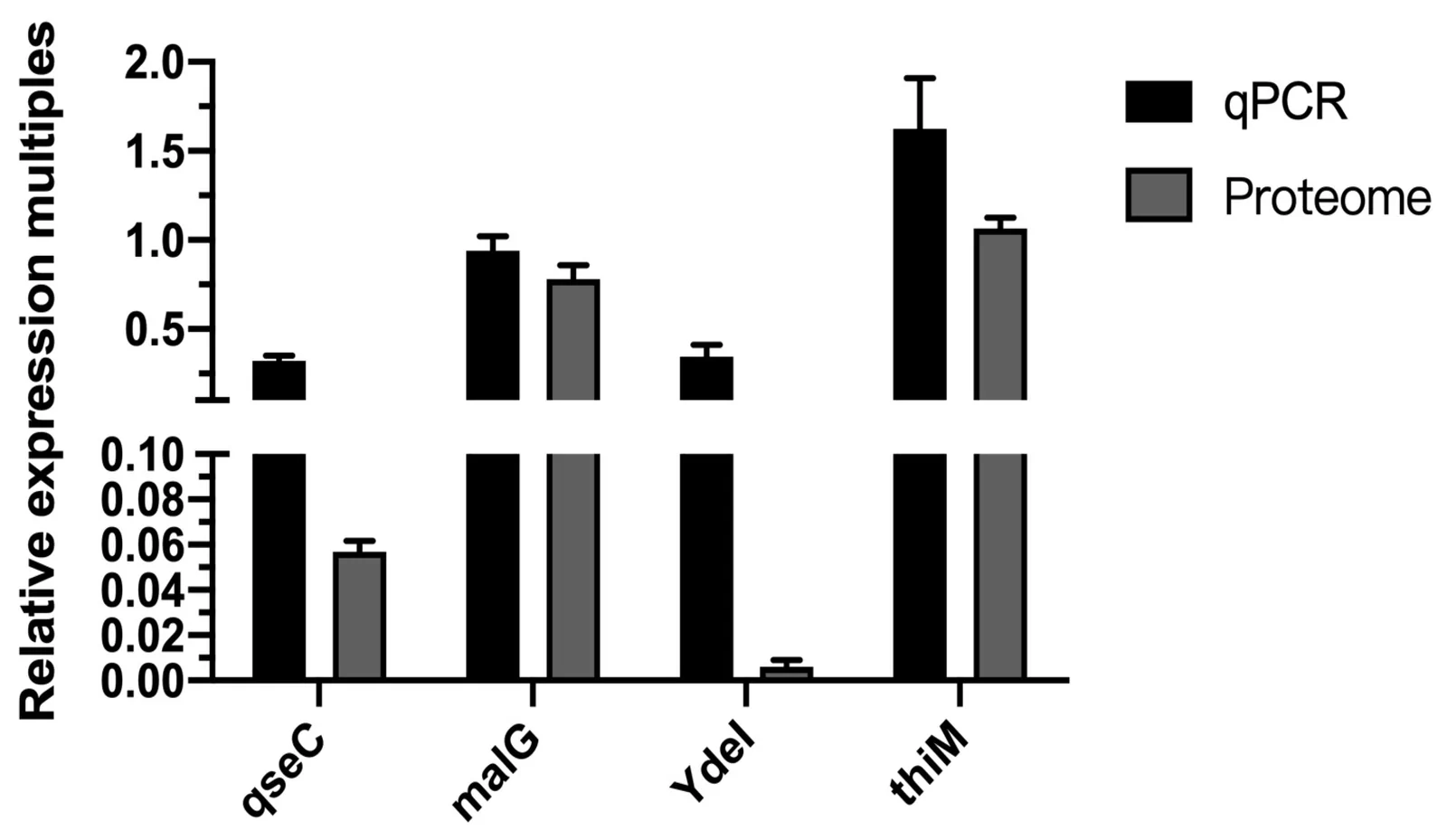
Validation of 4 DGEs by qRT-PCR. Data are shown as mean ± SEM.

To validate the proteomic findings, four differentially expressed genes (*qseC*, *malG*, *YdeI*, and *thiM*) were randomly selected and analyzed by qRT-PCR. The qRT-PCR results were highly consistent with the expression trends observed in the proteomic data (Figure 5), confirming the upregulation or downregulation of these genes. Minor discrepancies in the magnitude of expression changes between the two techniques may be attributed to differences in their sensitivity to detect gene expression dynamics.

### 3.6. Transcriptome and Proteome Correlation Analysis

**Figure 6:**
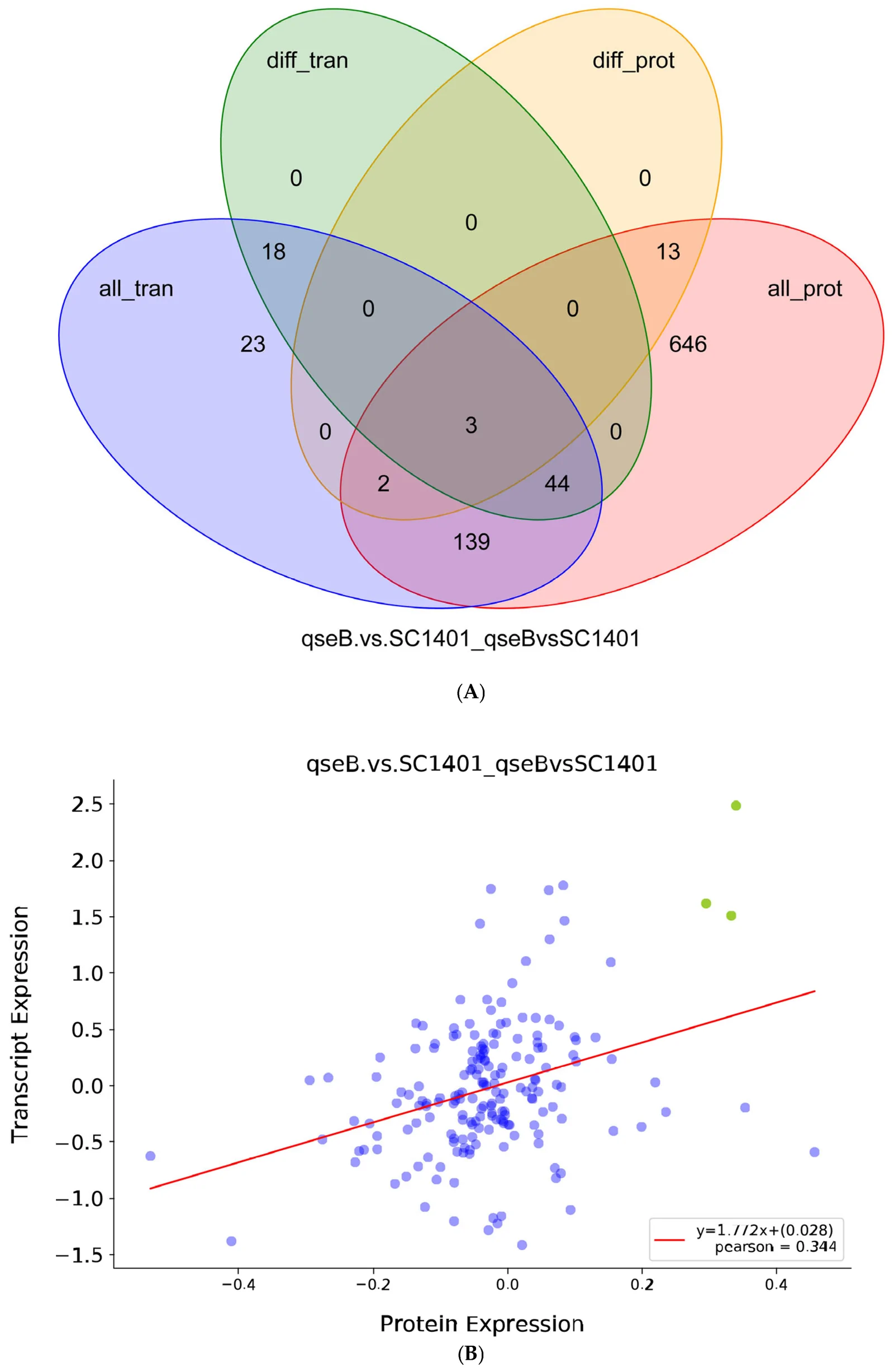

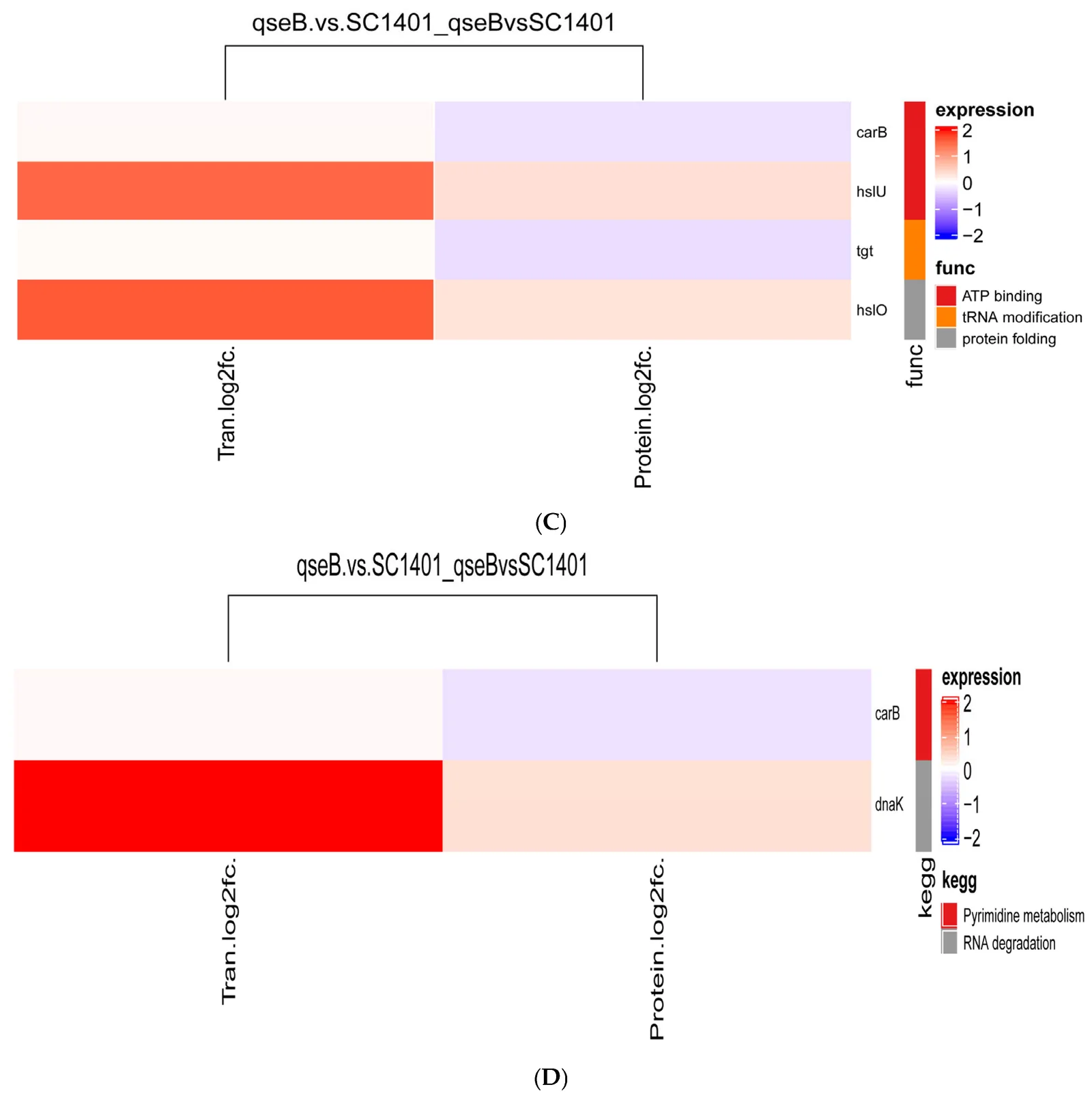
Correlation analysis between transcriptome and quantitative proteome data. (**A**) Venn diagram of transcriptome and proteome expression regulation. “All_tran” refers to all genes identified by transcriptomics. “Diff_tran” refers to differentially expressed genes from transcriptomics. “All_det” refers to all proteins identified by proteomics. “Diff_det” refers to differentially expressed proteins from proteomics. The diagram shows common and unique genes or proteins for each group. (**B**) Correlation analysis of transcriptome and proteome expression levels. Each point represents one protein. Green points indicate significantly differentially expressed proteins, and blue points indicate proteins without significant expression differences. The horizontal axis shows the log2 fold change in protein from proteomic data, and the vertical axis shows the log2 fold change in the corresponding genes from the transcriptomic data. (**C**) Clustering heatmap of GO functional enrichment. Red indicates upregulation, and blue indicates downregulation. The horizontal clustering groups proteins (genes) with similar expression patterns at both the proteomic and transcriptomic levels. Differential entries are based on protein data. (**D**) Clustering heatmap of KEGG pathway enrichment. Red indicates upregulation, and blue indicates downregulation. The horizontal clustering groups proteins (genes) with similar expression patterns at both proteome and transcriptome levels. Differential entries are based on protein data. For the clustering heatmaps in (**C**) and (**D**), a fold change (FC) > 1.5 was used to emphasize the most pronounced changes shared between the two omics datasets, whereas the proteome-wide DEP screening used FC > 1.2 to maximize coverage of biologically relevant changes.

Transcriptomic mRNA data from a previous study were integrated with the current proteomic data. Common targets were compared and visualized using a Venn diagram (Figure 6A). The analysis revealed 71 proteins identified by both transcriptomic and proteomic approaches, among which three genes (*dnaK*, *hslU*, and *hslO*) were differentially expressed in both omics layers. Further correlation analysis was performed on the fold changes in these shared genes (proteins) between the two omics datasets, as shown in Figure 6B.

To compare the functional features of differentially expressed proteins between SC1401 and Δ*qseB*, we used a fold change >1.5 and *p* < 0.05 (ANOVA) as screening criteria. This higher threshold was used for cross-omics visualization to highlight the most robustly changed genes/proteins shared between the two omics layers. It differs from the FC > 1.2 cutoff used for initial DEP screening in the proteomic dataset: FC > 1.2 provides broad coverage of biologically relevant proteins, whereas FC > 1.5 focuses on the most pronounced changes. We then performed GO enrichment analysis of these proteins with the gene expression levels from transcriptome data. The results are shown as a heatmap clustered by expression changes at the protein or gene level (Figure 6C; Table S9). A similar heatmap was generated for KEGG pathway enrichment and the corresponding transcript expression (Figure 6D).

## 4. Discussion

Transcriptomics and proteomics are essential tools for analyzing cellular physiological states and functional mechanisms. Integrating transcriptome and proteome data helps uncover gene regulatory mechanisms at the post-transcriptional level, and combining both approaches has become a fundamental strategy in systems biology research. In our previous study, we constructed ΔqseB, ΔqseC, and ΔqseBC mutant strains of G. parasuis. We previously demonstrated that the QseB/QseC system is involved in regulating biofilm formation, drug sensitivity, and response to environmental stress [14,15,16]. Scanning electron microscopy revealed major morphological changes in the ΔqseB mutant, in which most cells appeared shrunken and were coated with surface secretions or cellular debris. Transmission electron microscopy further showed damage to the cell wall and membrane, along with separation between the cytoplasm and cell wall. These results suggest that the absence of qseB may compromise bacterial structural integrity. Notably, whereas QseC deletion primarily affected lipopolysaccharide (LPS) biosynthesis and glycerolipid metabolism, QseB deletion was uniquely enriched in RNA degradation and pyrimidine metabolism pathways, suggesting distinct regulatory scopes of the sensor kinase and response regulator.

In this study, DIA-based quantitative proteomics was performed on six samples, and a total of 1493 proteins were identified. Using a threshold of fold change (FC) > 1.2 for upregulated proteins and <0.83 for downregulated proteins with *p* < 0.05, we identified 55 significantly differentially expressed proteins. Among these, 24 were upregulated and 31 were downregulated. These results suggest that QseB may regulate the expression of many proteins and acts mainly as a positive regulator. The upregulated proteins in the Δ*qseB* mutant included RaiA, DnaK, LeuA, HslV, HtpG, HslO, RsfS, RluA, HslU, GroL, RlmE, and ClpB. RaiA inhibits protein translation by binding to aminoacyl-tRNA [18]. Disruption of its non-coding RNA (the *raiA* motif) significantly affects sporulation and cell aggregation [19].

The ribosome silencing factor RsfS binds to the uL14 protein and inhibits ribosome subunit assembly [20]. It also helps maintain energy homeostasis under nutrient stress [21]. The molecular chaperones DnaK (Hsp70) and GroEL (Hsp60) are highly conserved [22]. DnaK assists in the folding of newly synthesized polypeptide chains, and GroEL maintains protein stability [23,24]. DnaK exhibits functional conservation across species, even in psychrophilic bacteria [25]. The heat shock protein HtpG is involved in stress response [26]. It also directly regulates host infection in *Salmonella typhimurium* [27]. The protease complex HslVU has two ATP-dependent functions, with the HslV subunit cleaving peptide bonds and HslU providing ATPase activity [28]. Protease activity can also be activated by peptides derived from the C-terminus [29]. The antimicrobial peptide LeuA [30] targets the surface of Gram-positive bacteria such as *Listeria monocytogenes* [31]. The chaperone ClpB helps pathogens resist host oxidation and heat stress by degrading toxic proteins, thereby facilitating infection [32]. HslO can repair DNA damage in Δ*recA polA* cells and reduce growth inhibition [33]. In metabolic regulation, RluA functions as a pseudouridine synthase that catalyzes mRNA modification [34]. Deletion of the *lpb* gene interferes with glucose metabolism and disrupts the synthesis of amino acids and lactate [35]. Proteins downregulated in Δ*qseB* include LolB, YkgO, Tgt, ThiL, QseC, and CarB. LolB acts as an outer membrane-anchored lipoprotein and catalyzes the final step in transferring lipoproteins from the inner to the outer membrane in *Escherichia coli* [36]. Both LolA and LolB are highly conserved in the phylum Bacteroidetes. They play essential roles in bacterial gliding and the type IX secretion system (T9SS) [37]. ThiL is a key enzyme in thiamine biosynthesis and the salvage pathway in *Pseudomonas aeruginosa*. It may serve as a potential antibacterial target [38]. Differentially expressed proteins in the Δ*qseB* mutant were identified by GO analysis of the DIA quantitative proteomics data. The main enriched GO terms included protein folding, tRNA modification, unfolded protein binding, macromolecule modification, receptor activity, methylation, RNA modification, outer membrane localization, cell communication, tRNA wobble position uridine thiolation, 2-isopropylmalate synthase activity, leucine biosynthetic process, threonine-type endopeptidase activity, proteasome core complex, proteolysis involved in cellular protein catabolism, cellular response to starvation, pathogenesis, and cell outer membrane function. Further integration of transcriptomic and proteomic data through comparative GO analysis revealed that the regulatory function of QseB is primarily associated with pathways related to ATP binding, tRNA modification, and protein folding.

KEGG pathway enrichment analysis showed that differentially expressed proteins identified by DIA-based quantitative proteomics were significantly enriched in the following pathways: RNA degradation, the two-component system, ascorbate and aldarate metabolism, thiamine metabolism, valine, leucine and isoleucine biosynthesis, sulfur relay system, phosphotransferase system, alanine, aspartate and glutamate metabolism, 2-oxocarboxylic acid metabolism, pyruvate metabolism, quorum sensing, pyrimidine metabolism, ABC transporters, and ribosome. Integration of the proteomic data with previously published transcriptome data revealed that both the differentially expressed proteins and their corresponding genes were significantly enriched in pyrimidine metabolism and RNA degradation pathways. The core stress response proteins identified by DIA quantitative proteomics included: molecular chaperones GroEL and Hsp70, protease components HslU and HslV, heat shock proteins ClpB, HslO, and RrmJ, translation regulatory factors RsfS and RluA, and the quorum sensing protein QseC. Among these, the highly conserved methyltransferase RrmJ catalyzes methylation of the U2552 site in the 23S rRNA A-loop at the 2′-O-ribose position [39]. Loss of RrmJ function leads to impaired growth, reduced translation rates, and decreased stability of the 70S ribosome [40].

While both QseB and QseC belong to the same two-component system, our comparative analysis reveals that their deletion leads to distinct proteomic signatures. In the Δ*qseC* mutant, label-free quantitative proteomics identified 39 differentially expressed proteins (DEPs), with upregulation of PlsB (glycerol-3-phosphate acyltransferase) and downregulation of UvrA (DNA repair), HldD (LPS biosynthesis), and HsdR (defense mechanism). In contrast, the Δ*qseB* mutant exhibited 55 DEPs, with a prominent upregulation of heat shock proteins (DnaK, GroEL, HtpG), protease components (HslV, HslU, ClpB), and translation inhibitors (RaiA, RsfS). Strikingly, none of the DEPs were shared between the Δ*qseC* and Δ*qseB* mutants at the protein level. This suggests that the sensor kinase QseC and the response regulator QseB control largely non-overlapping protein networks under standard growth conditions. We propose two hypotheses to explain the largely non-overlapping protein networks controlled by QseB and QseC. On one hand, QseC only functions as an extracellular signal sensor with unique phosphatase activity to suppress QseB activity, merely regulating proteins linked to membrane and environmental adaptation. In contrast, QseB can be phosphorylated without QseC and independently governs metabolism and proteostasis pathways, leading to intrinsically different target repertoires. On the other hand, loss of QseC eliminates its dephosphorylation restraint, and non-cognate PmrB continuously activates QseB through robust cross-talk. Persistent phosphorylated QseB triggers a series of regulatory cascades irrelevant to QseC, further widening the difference in their downstream protein profiles [41].

While *dnaK* was downregulated in the Δ*qseC* transcriptome [10], it was upregulated in the Δ*qseB* proteome, suggesting that QseC and QseB may exert opposing transcriptional control over certain chaperone genes. The unique upregulation of translation inhibitors (RaiA, RsfS) and the HslVU protease complex in the Δ*qseB* reflects a stress survival strategy centered on energy conservation and toxic protein clearance, which is distinct from the “offensive–defensive shift” (lipid storage vs. defense downregulation) observed in the Δ*qseC*. Together, these findings indicate that QseB orchestrates a stress adaptation program fundamentally different from that of its cognate sensor QseC. We observed a striking upregulation of multiple molecular chaperones (DnaK, GroEL, HtpG, HslO, and GroES) in the Δ*qseB* mutant. This robust induction of chaperones is likely more than a passive stress marker; a marked imbalance in chaperone abundance can interfere with cellular polypeptide folding kinetics, whereby excess chaperone molecules may competitively bind nascent polypeptides, redirect normal folding pathways, and cause aberrant exposure of hydrophobic residues, thereby generating non-optimal protein–protein interactions [42]. A growing body of studies in eukaryotic systems has demonstrated that Hsp90 chaperones (the bacterial ortholog of which is HtpG) act as central hubs of protein interaction networks, and functional disruption of these chaperones leads to widespread interactome rewiring. We speculate that a similar scenario occurs in bacteria, in which chaperone imbalance triggers aberrant protein interactions that compromise network robustness, and these proteostasis perturbations may propagate across the interactome and further reshape the global bacterial gene regulatory network (GRN). For example, altered GroEL/S abundance has been shown to directly modulate σ^32^ activity and reprogram the heat-shock transcriptional landscape [43]. Collectively, this network-wide disturbance, combined with the cell envelope structural defects and impaired stress resistance observed in the Δ*qseB* mutant, suggests that the attenuated environmental adaptability and pathogenicity of the mutant strain may result from concerted dysregulation between proteostasis networks and gene regulatory networks. This phenotypic alteration is consistent with previous findings that deletion of the QseB/QseC two-component system impairs bacterial stress tolerance and virulence [44]. Although this mechanistic model remains speculative, it is well supported by existing studies on proteostasis and chaperone networks.

The primary goal of this study was to compare global proteomic profiles between the wild-type strain and the Δ*qseB* mutant, focusing on network-level alterations rather than single-gene functional validation. For this reason, multi-omics analyses using the complemented strain were not performed in the present work. Our group has previously constructed and functionally validated the Δ*qseB*-complemented strain in transcriptomic studies [17]. Furthermore, animal experiments with the QseBC double-deletion mutant and its complemented strain confirmed that phenotypic defects such as reduced adhesion and virulence are specifically caused by the loss of QseB, excluding confounding effects from random genomic mutations [14]. Moreover, earlier transcriptomic data also demonstrated significant upregulation of molecular chaperones including DnaK and GroEL following *qseB* deletion, which is highly consistent with our proteomic observations and indirectly supports the inference that proteostasis imbalance remodels the regulatory network. Our previous work on the QseBC double-knockout mutant [14] showed that QseBC deletion significantly reduced adhesion to and invasion of PAM cells, confirming the importance of this system in host–pathogen interactions. The present study was designed to perform global proteomic and morphological characterization of the Δ*qseB* mutant. Given that functional activity in host cells is distinct from proteomic profiling, further investigation of the mutant in cell-based models would provide complementary evidence regarding its biological relevance. Cell-based studies, together with the generation of a complemented strain and targeted interactome analysis, will be undertaken in future work to further verify the regulatory network identified here.

## 5. Conclusions

This study integrated proteomic and previously obtained transcriptomic data to reveal the stress response mechanisms in the *G. parasuis* Δ*qseB* mutant. Electron microscopy showed that the loss of *qseB* led to damage to the cell wall and membrane, cell shrinkage, and cytoplasmic leakage, indicating that this gene is essential for maintaining cellular structural integrity. DIA-based quantitative proteomics identified 55 significantly expressed proteins (24 upregulated, 31 downregulated). The upregulated proteins included translation inhibitors (RaiA and RsfS), which conserve energy by blocking aminoacyl-tRNA binding and ribosome assembly, as well as chaperones (DnaK, GroEL, and HtpG) that maintain protein stability and stress defense, and proteases (HslVU and ClpB) involved in the degradation of toxic proteins. The modification enzyme RrmJ, which methylates the U2552 site of 23S rRNA, was also affected, and its loss led to impaired growth. Pathway analysis showed that these proteins participate in pyrimidine metabolism, RNA degradation, ATP binding, tRNA modification, and protein folding. Together, these results suggest that QseB acts as a global regulator, coordinating a stress response network that influences bacterial adaptation to the environment. However, its deeper roles in biofilm formation and virulence regulation warrant further investigation.

## Figures and Tables

**Table 1:** Primers used for qRT-PCR validation of proteomic results.

GenBank ID	Primer	Sequence (5′–3′)	Length (bp)
A4U84_02995	16SrRNA-F	CCACCTGCCATAAGATGAGC	122
A4U84_02995	16SrRNA-R	GGACCGTGTCTCAGTTCCAG	122
*thiM*	*thiM*-F	CGTGCCTGTGGTGCTTGAC	113
*thiM*	*thiM*-R	CGCCAGCATTACCACGAATAGC	113
*YdeI*	*YdeI*-F	TCACGGTGGCTATCACGATGG	112
*YdeI*	*YdeI*-R	GGTGTCATCAGTTGCTTTAAGTGC	112
*malG*	*malG*-F	CGGGCAACTTGGCATTAGGG	113
*malG*	*malG*-R	TGGCGGCGGAGTAACAGTG	113
*qseC*	*qseC*-F	TCGTGAGCAGCGTCAGAGG	115
*qseC*	*qseC*-R	CGACAGCAGTGCCGAAAGAG	115

Primers used for qRT-PCR validation of proteomic data. GenBank ID: gene identifier in the NCBI GenBank database; Primer: primer name; Sequence (5′–3′): primer sequence; Length (bp): amplicon length.

## Data Availability

The original contributions presented in this study are included in the article/Supplementary Material. Further inquiries can be directed to the corresponding author.

## Supplementary Materials

The following supporting information can be downloaded at: https://www.mdpi.com/article/10.3390/microorganisms14092073/s1, Figure S1: Quality assessment of proteomic data; Table S1: Protein Quantification Results; Table S2: Protein Differential Analysis Results; Table S3: Detailed Information of Protein Differential Analysis; Table S4: Data for Volcano Plot of Protein Differential Expression; Table S5: GO Enrichment Results of Upregulated Proteins; Table S6: GO Enrichment Results of Downregulated Proteins; Table S7: KEGG Enrichment Results; Table S8: Hub Proteins Identified by the MCC Algorithm; Table S9: Correlation Analysis Results of Commonly Identified Genes (Proteins) from Transcriptomic and Proteomic Data.
