# Supplementary material for: Proteomic Profiling Reveals Intracellular Regulatory Network Disturbance Induced by QseB Deletion in Glaesserella parasuis
Source: Microorganisms. 2026 Sep 16;14(9):2073. doi: 10.3390/microorganisms14092073 (PMC13608904; doi:10.3390/microorganisms14092073)
Supplement: Supplementary file 1 [file microorganisms-14-02073-s001.zip › microorganisms-4548650-supplementary.pdf]

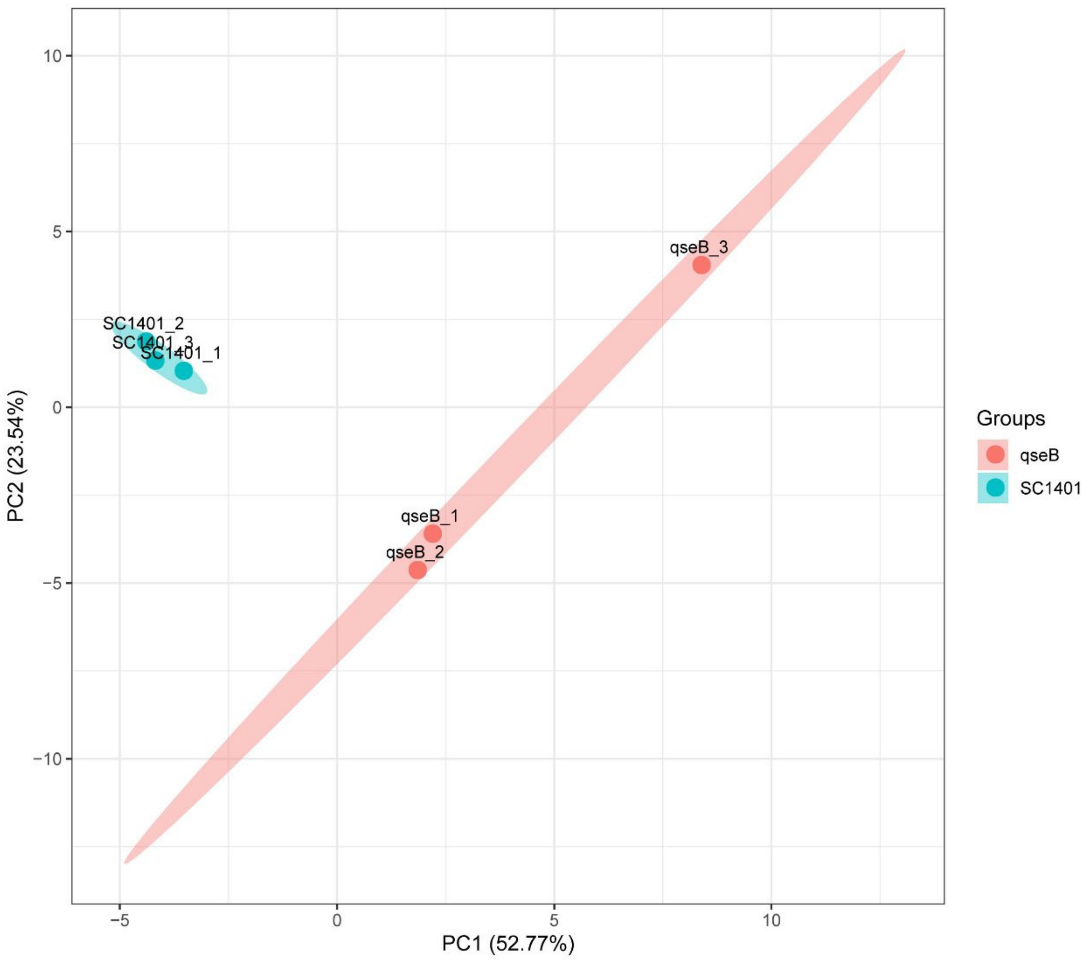

10
5
0
−5
−10
PC2 (23.54%)
−5
0
5
10
PC1 (52.77%)
SC1401_2
SC1401_3
SC1401_1
qseB_3
qseB_1
qseB_2
Groups
qseB
SC1401

(A)

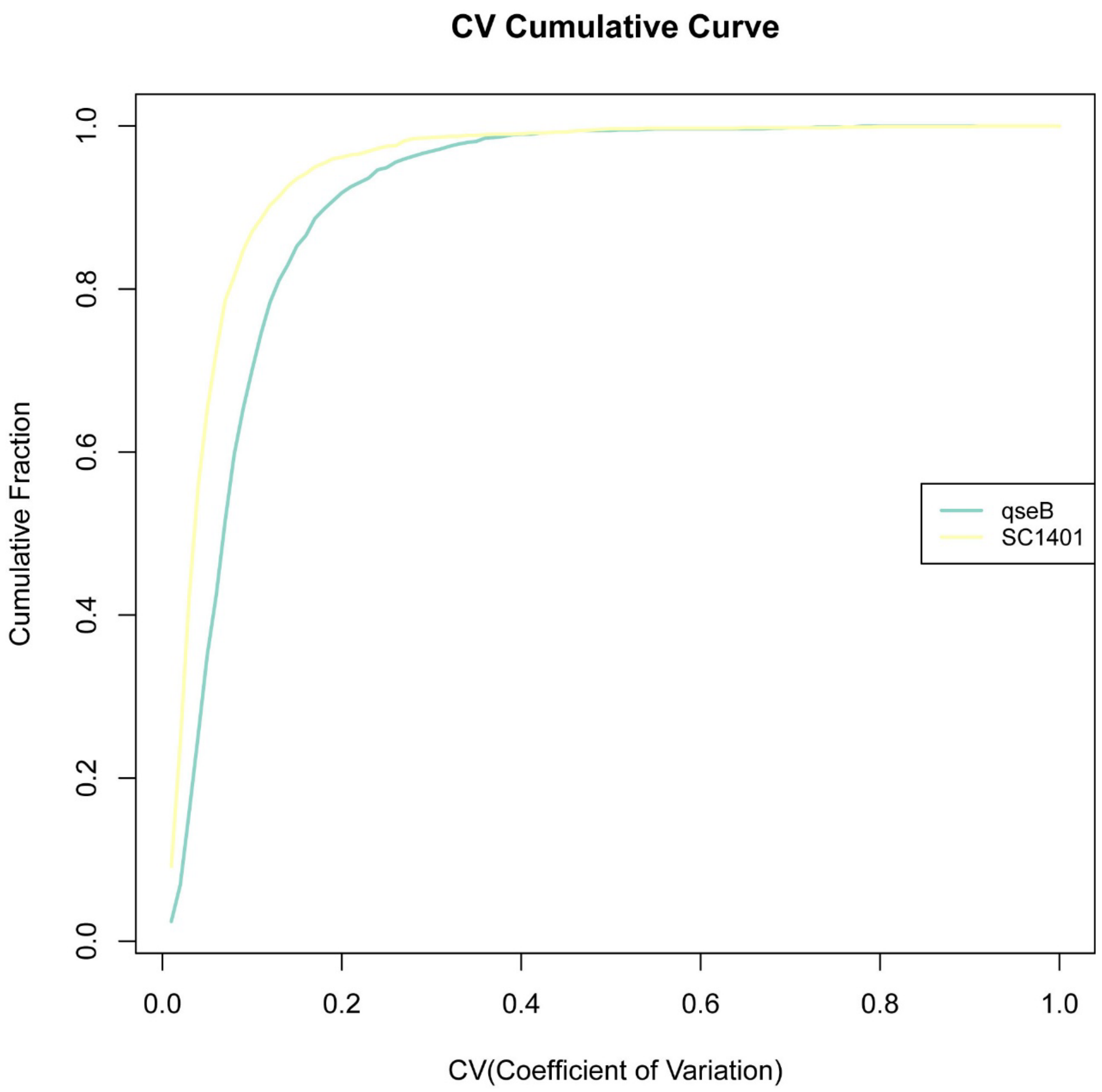

(B)

Figure S1. Quality assessment of proteomic data. (A) Principal component analysis (PCA) showing global differences in protein expression among samples and intragroup variability. (B) Distribution of the coefficient of variation (CV) used to evaluate data dispersion and experimental reproducibility.

Table S1. Protein Quantification Results. Protein： Protein: protein identifier in the database; Description: description of the protein in the database; Gene: name of the gene corresponding to the protein in the database; the remaining columns contain the relative quantification values for each sample. The complete dataset is provided as a separate Excel file (Table S1).

Table S2. Protein Differential Analysis Results. Compared Samples: the pair of

samples being compared, with the former compared against the latter; Num. of Total Quant.: the number of proteins commonly identified in both sample groups (proteins not identified in both groups cannot be assigned as up- or downregulated); regulated Type: regulation type of the protein; fold-change: fold-change threshold.

Table S3. Detailed Information of Protein Differential Analysis. Protein: identifier of the identified protein sequence; Description: protein description; Gene: gene name corresponding to the protein; FC: fold change of protein expression between the two groups; *P* value: *p* value from the significance test between the two groups; log2FC: log2-transformed fold change; UP.DOWN: upregulation or downregulation of the protein; GO_NUM: number of GO terms matched to the protein in the GO database; GO_Description: detailed description of all matched GO terms; KEGG_Description: description of the KEGG ortholog (KO); KEGG_EC: corresponding EC number in the KEGG database; KEGG_ko: pathway information involving the protein in the KEGG database; COG_Function_Description: functional description annotated in the COG database; COG_Function_Class: functional class to which the COG function belongs; COG_Class_Description: description of the COG functional class.

Table S4. Data for Volcano Plot of Protein Differential Expression. Protein: identifier of the identified protein sequence; Description: protein description; Gene: name of the gene corresponding to the protein; the columns after Gene contain the expression values of differentially expressed proteins across different samples.

Table S5. GO Enrichment Results of Upregulated Proteins. GO_ID: GO identifier; GO_Term: description of the GO term; GO_Class: GO category (MF, CC, or BP); *P* value: *p* value from the enrichment analysis; Adjusted Pv: adjusted *p* value; *x*: number of differentially expressed proteins associated with the GO term; *y*: number of background (all) proteins associated with the GO term; *n*: number of differentially expressed proteins annotated with GO terms; *N*: number of background (all) proteins annotated with GO terms; GO levl: hierarchical level of the GO term in the GO database; Prot ID: protein identifier.

Table S6. GO Enrichment Results of Downregulated Proteins. GO_ID: GO identifier; GO_Term: description of the GO term; GO_Class: GO category (MF, CC, or BP); *P* value: *p* value from the enrichment analysis; Adjusted Pv: adjusted *p* value; *x*: number of differentially expressed proteins associated with the GO term; *y*: number of background (all) proteins associated with the GO term; *n*: number of differentially expressed proteins annotated with GO terms; *N*: number of background (all) proteins annotated with GO terms; GO levl: hierarchical level of the GO term in the GO database; Prot ID: protein identifier.

Table S7. KEGG Enrichment Results. MapID: identifier of the enriched KEGG pathway; Map Title: name of the enriched KEGG pathway; *P* value: *p* value from the enrichment analysis; Adjusted Pv: adjusted *p* value; *x*: number of differentially expressed proteins associated with the pathway; *y*: number of background (all) proteins associated with the pathway; *n*: number of differentially expressed proteins annotated with KEGG terms; *N*: number of background (all) proteins annotated with KEGG terms; Prot ID: list of proteins enriched in the pathway.

Table S8. Hub Proteins Identified by the MCC Algorithm. Rank: ranking of the hub proteins based on the MCC score; Name: protein name; Score: MCC score calculated by the cytoHubba plugin. A higher score indicates higher connectivity and a more central position of the protein in the network.

Table S9. Correlation Analysis Results of Commonly Identified Genes (Proteins) from Transcriptomic and Proteomic Data. Gene_id: gene name; Tran_id: transcript ID corresponding to the gene; Prot_id: protein ID corresponding to the gene; GO_fun: significantly enriched GO terms; kegg: significantly enriched KEGG pathways; Protein(log2fc): log2-transformed fold change of the significantly differentially expressed protein corresponding to the gene; Protein (pval): *p* value of the significantly differentially expressed protein corresponding to the gene; Tran(log2fc): log2-transformed fold change of the significantly differentially expressed transcript corresponding to the gene; Tran (pval): *p* value of the significantly differentially expressed transcript corresponding to the gene.
